# Injectable and Self‐Curing Single‐Component Hydrogel for Stem Cell Encapsulation and In Vivo Bone Regeneration

**DOI:** 10.1002/advs.202304861

**Published:** 2024-02-14

**Authors:** Seo Young Cheon, Ji Sun Park, Yeeun Lee, Chaehyun Lee, Hayoung Jeon, Donghyun Lee, Se Hee Kim, Seong Gi Lim, Heebeom Koo

**Affiliations:** ^1^ Department of Medical Life Sciences Department of Biomedicine & Health Sciences, and Catholic Photomedicine Research Institute College of Medicine The Catholic University of Korea 222 Banpo‐daero, Seocho‐gu Seoul 06591 Republic of Korea

**Keywords:** bone regeneration, hyaluronic acid, injectable hydrogel, phenylboronic acid, spermidine, stem cell

## Abstract

An ideal hydrogel for stem cell therapy would be injectable and efficiently promote stem cell proliferation and differentiation in body. Herein, an injectable, single‐component hydrogel with hyaluronic acid (HA) modified with phenylboronic acid (PBA) and spermidine (SM) is introduced. The resulting HAps (HA‐PBA‐SM) hydrogel is based on the reversible crosslinking between the diol and the ionized PBA, which is stabilized by the SM. It has a shear‐thinning property, enabling its injection through a syringe to form a stable hydrogel inside the body. In addition, HAps hydrogel undergoes a post‐injection “self‐curing,” which stiffens the hydrogel over time. This property allows the HAps hydrogel to meet the physical requirements for stem cell therapy in rigid tissues, such as bone, while maintaining injectability. The hydrogel enabled favorable proliferation of human mesenchymal stem cells (hMSCs) and promoted their differentiation and mineralization. After the injection of hMSCs‐containing HAps into a rat femoral defect model, efficient osteogenic differentiation of hMSCs and bone regeneration is observed. The study demonstrates that simple cationic modification of PBA‐based hydrogel enabled efficient gelation with shear‐thinning and self‐curing properties, and it would be highly useful for stem cell therapy and in vivo bone regeneration.

## Graphical abstract

An injectable, single‐component hydrogel is developed based on the reversible crosslinking between the diol and the ionized phenylboronic acid, which is stabilized by spermidine. It has a shear‐thinning property and undergoes a post‐injection “self‐curing,” which stiffens it over time. These properties allow it to meet the physical requirements for stem cell therapy in rigid tissues like bone, while maintaining injectability.

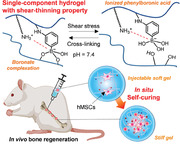

## 1 Introduction

A hydrogel is a 3D network containing water that has been used for various biomedical applications, including as a drug depot, filler, adhesive, and coating material.^[^ ^1^ ^]^ In particular, hydrogel‐based cell scaffolds have been reported to greatly increase the efficacy of cell therapy, and thus have been intensely studied.^[^ ^2^ ^]^ For this purpose, injectable hydrogels that are in the sol state outside of the body and that undergo gelation inside the body are highly desirable because they can be injected with syringe needles in a minimally invasive manner.^[^ ^3^ ^]^ In addition, such hydrogels can adapt their shape according to the injection site, providing an exact fit. This feature is helpful for filling irregular defects and improving the therapeutic effect. Thermosensitive hydrogels and crosslinkable polymers are representative injectable hydrogels.^[^ ^4^ ^]^

For a long time, many studies have reported that the physical properties, particularly the stiffness of the surrounding environment, have a great influence on the long‐term fate of injected therapeutic cells.^[^ ^5^ ^]^ The stiffness of hydrogel scaffold can affect the adhesion, proliferation, and differentiation lineages of the cells within.^[^ ^6^ ^]^ In particular, the effect of stiffness is more important when it is applied to the regeneration of highly rigid tissues, like bone.^[^ ^7^ ^]^ Therefore, the ideal injectable hydrogel will be fluid enough to pass through syringe needles, but still achieve high stiffness inside the body. Thermal gelation focusing on the temperature change during injection is useful, and thermosensitive polymers like poly(N‐isopropylacrylamide) and Pluronic have been used for this purpose.^[^ ^8^ ^]^ However, the physical property changes enabled by body temperatures are generally not sufficient, so it is difficult to meet this special requirement. In the case of cross‐linkable polymers, most are based on physical or chemical crosslinking between polymers creating networks and enabling gelation.^[^ ^9^ ^]^ Various bindings including charge–charge interaction, avidin–biotin, cyclodextrin–adamantane, and click chemistry have been used for such hydrogels.^[^ ^10^ ^]^ In these hydrogels, each polymer chain is prepared in aqueous solution, but they became stiff after mixing and crosslinking. This type of hydrogel typically uses dual‐barrel syringes that mix two solutions during injection or is injected right after mixing in the short time before complete gelation occurs.^[^ ^11^ ^]^ However, these gelation processes are too sensitive, so that the property of hydrogel can be changed depending on the person who inject and gelation inside the needle is also possible.

From this perspective, hydrogels equipped with an intrinsic shear‐thinning property is highly useful for injection into the human body. Most of such hydrogels show reversible bond breaking and re‐formation.^[^ ^12^ ^]^ This process can enable fluidic movement in a syringe needle under high pressure, even though the hydrogel stiffens again after injection and when it is free from high pressure inside body. Generally, hydrogels based on dopa or gallol groups shows such behavior, which enables facile injection without careful attention, unlike hydrogels based on the irreversible crosslinking.^[^ ^13^ ^]^ Supramolecular assembly with cyclodextrin or cucurbiturils also provided reversible and non‐covalent binding and “self‐healing” property. Recent studies showed its usefulness to develop injectable hydrogels.^[^ ^14^ ^]^ However, the rigidity of the hydrogels obtained after injection is still insufficient for bone regeneration in many such cases.

**Scheme 1:**
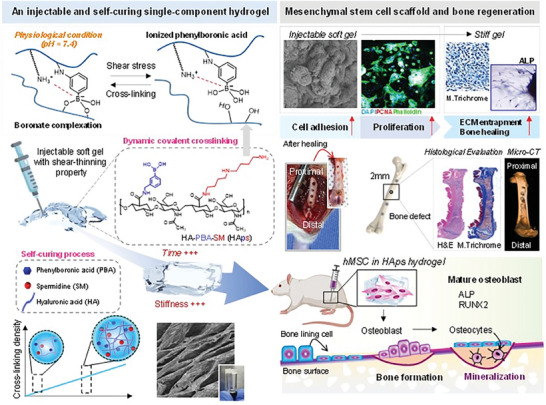
Schematic illustration of the injectable and self‐curing single‐component hydrogel and its application for stem cell therapy and in vivo bone regeneration.

In this study, we develop an injectable, single‐component hydrogel capable of in situ self‐curing based on hyaluronic acid (HA) modified with phenylboronic acid (PBA) and spermidine (SM) (**Scheme** **1**). These polymer chains are crosslinked via a reversible, dynamic covalent bond between diols in HA and PBA, which is stabilized by the amine groups in SM. The resulting hydrogel possesses a shear‐thinning property that enables injection via a syringe, and it is a suitable scaffold for human mesenchymal stem cells (hMSCs). In particular, this hydrogel becomes stiffer over time due to increasing crosslinking. This self‐curing process is useful for meeting the mechanical requirements for cell therapy in rigid tissues like bone. Importantly, it is meaningful that this hydrogel fully achieves these properties with only a single component, not a mixture of two or more materials. After development and analysis of the rheological properties of this hydrogel, we evaluate its potential as a practical scaffold for hMSCs by observing hMSC proliferation, aggregate formation, extracellular matrix (ECM) mineralization, and osteogenic differentiation. Finally, we apply this system to a rat femoral defect model and monitor the efficacy of stem cell therapy for in vivo bone regeneration. We expect that our hydrogel would prevent stem cells escaping form the target disease area and facilitate their osteogenic differentiation and mineralization for bone repair with help of the gradually increased stiffness.

## 2 Results and Discussion

### 2.1 Development and Characterization of HAps Hydrogel

**Figure 1:**
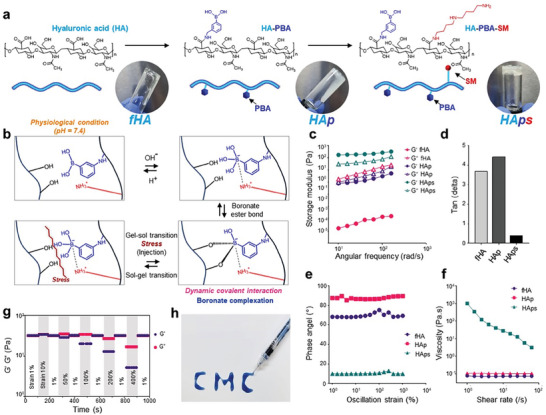
Development and characterization of HAps hydrogel. a) Synthetic scheme and chemical structure of HAps and photographic images of HAps hydrogel. b) Crosslinking mechanism between diols and ionized PBA stabilized by amine groups of SM for reversible gel–sol transition by stress, providing the shear‐thinning property. c) Angular frequency‐modulus (G′,G″) plots of free HA (fHA), HA‐PBA (HAp), and HA‐PBA‐SM (HAps). d) HAps hydrogel tan(delta) values at 10 rad s^−1^. e) Oscillation strain‐phase angle of HAps hydrogel. f) Shear rate‐viscosity correlation of HAps hydrogel obtained by rheology analysis of rotation. g) Shear‐thinning property of HAps hydrogel demonstrated by the continuous step strain measurements to 400%. h) Photographic image of HAps hydrogel injected by 31‐gauge syringe needle. Trypan blue was added for color and CMC means Catholic Medical Center.

The synthetic scheme of the HA polymer modified with PBA and SM (HAps) is shown in **Figure** **1**a. First, PBA was conjugated to the carboxylic acids in HA via amide coupling reaction. The degree of substitution (DS) of PBA was analyzed using an alizarin red S assay, and 35% was the maximum DS value which was used in following studies (Table S1, Supporting information). Then, SM, a natural, small chemical with amine groups, was similarly conjugated to the remaining carboxylic acids of HA‐PBA via amide bonds. We observed aggregation when the spermidine conjugation reaction was carried out for longer than 24 h or at a higher molar ratio than 1.5:1 (SM:the remaining carboxylic acids in HA‐PBA), which defined the final experimental conditions used (Table S2, Supporting information). The gelation mechanism of HAps is a reversible dynamic conjugation between the diol group of HA and PBA, which generates boronate ester (Figure 1b). The tetrahedral structure of the ionized PBA is more reactive than its original form.^[^ ^15^ ^]^ In HAps, the amine groups of SM can stabilize this ionized PBA‐containing negative charge via electrostatic interaction. Consequently, the amount of the ionized PBA increases in HAps compared to HAp without SM, and this increase is beneficial for crosslinking with diols. This mechanism is supported by the fact that HAps enabled gelation at a 3% (w/w) ratio, which does not trigger gelation when using the control HAp without SM. The crosslinking between diol and PBA is reported to be reversible: it can be broken down using external stress and recovered when the stress disappears.^[^ ^16^ ^]^ This property provides the shear‐thinning effect of HAps hydrogel.

A DHR‐10 rheometer was used to examine the rheology of HAps hydrogel (Figure 1c). Unlike hydrogels using free HA (fHA) without modification or HAp, the storage modulus (G′) is greater than the loss modulus (G″) in HAps hydrogels, indicating that only HAps forms stable hydrogel with sufficient crosslinking. The small tan(delta) = G″/G′ value and phase angle of HAps was observed in a wide range of oscillation strain (%), showing its high elasticity (Figure 1d,e). In the rotation test, HAps demonstrated a decrease in viscosity at a dependent shear rate, which is typical in shear‐thinning fluids (Figure 1f).^[^ ^17^ ^]^ A similar trend was observed in complex viscosity analysis (Figure S1, Supporting Information). In other words, due to the dynamic covalent bond, HAps has the fluidity of a gel–sol transition at high shear stress. In Figure 1g, HAps shows a gel‐sol transition (G″ > G′) under high strain and restoration to the original (G′ > G″) sol–gel transition under low strain, demonstrating its shear‐thinning property. Due to this property, HAps can pass through the narrow needle tip of 26‐gauge syringe under high shear stress (Figure 1h). In addition, we confirmed the decomposition behavior of fHA and HAps hydrogel at 6080 U mL^−1^ hyaluronidase.^[^ ^18^ ^]^ As expected, fHA was degraded by hyaluronidase and reached its half‐life before 24 h, while HAps hydrogel remained >70% even after about 1 week (Figure S2, Supporting information). This suggested that HAps hydrogel was stable for longer period compared to free HA polymer under condition like the in vivo environment. These data show the potential of HAps, with its shear‐thinning property, as an injectable hydrogel for clinical operations.

### 2.2 In Vitro Time‐Dependent Self‐Curing of HAps Hydrogel

**Figure 2:**
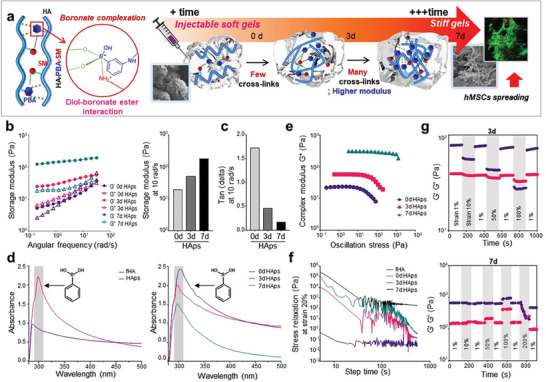
Self‐curing of HAps hydrogel in vitro. a) Schematic illustration of HAps hydrogel becoming stiffer over time. b) Angular frequency–modulus (G′,G″) plots of HAps hydrogels and comparison of storage modulus (G′) values at 10 rad s^−1^. c) Comparison of tan(delta) of hydrogels at 10 rad s^−1^. d) UV–vis spectra of fHA, HAp, and HAps hydrogel over time. e) Comparison of oscillation stress‐complex modulus at different times. f) Stress relaxation analysis of hydrogels at different time points. g) Shear‐thinning of hydrogels at different time points demonstrated by the continuous step strain measurements to 100 or 200%.

Interestingly, we observed that HAps hydrogel undergoes a self‐curing process and hardens over time after gelation, which would be beneficial for stem cell spreading and differentiation when applied as an artificial ECM in physiological environment for bone tissue (**Figure** **2**a). Figure 2b depicts the time‐dependent changes in the mechanical properties of HAps. The storage modulus of HAps gradually increases, reaching an 18‐fold increase compared to the initial state on day 7. The tan(delta) value of HAps at day 7 was near zero, which is close to that of an ideal elastic body (Figure 2c). We expect that the time‐dependent increase of the conjugation between diol and PBA caused this phenomenon. PBA has a distinct absorbance at 300 nm, which could be only observed in HAps, not in fHA without PBA (Figure 2d).^[^ ^19^ ^]^

We observed that the absorbance of PBA in HAps decreased over time due to participation in binding with diols, which was not observed in the solution containing only free PBA (Figure S3, Supporting information). Increased crosslinking would result in the increased hydrogel stiffness, as observed in the highest storage modulus was observed on day 7 with the lowest absorbance of PBA. The stiffness of HAps on day 7 was also indicated by the change in complex modulus with oscillation stress (Figure 2e). In comparison to the samples on day 0 and 3, HAps on day 7 exhibited the highest complex modulus and the least deformation, even under a high oscillation stress of about 1000 Pa. It is reported that cells exhibit completely different behaviors in soft and stiff environments.^[^ ^20^ ^]^ In a soft environment, cell migration and proliferation occur actively, whereas the differentiation of grown cells is more active in a stiff environment. In a three‐dimensional environment, most cells had a strain of about 20%.^[^ ^21^ ^]^ When a strain of 20% was applied to HAps on day 7, there was almost no difference in stress relaxation, indicating that it would be sufficiently stiff for cells (Figure 2f). Furthermore, HAps on day 7 becomes a near‐ideal elastomer to the extent that the shear‐thinning property is lost (Figure 2g).

### 2.3 Evaluation of hMSC Adhesion and Proliferation in HAps Hydrogel for 7 Days

**Figure 3:**
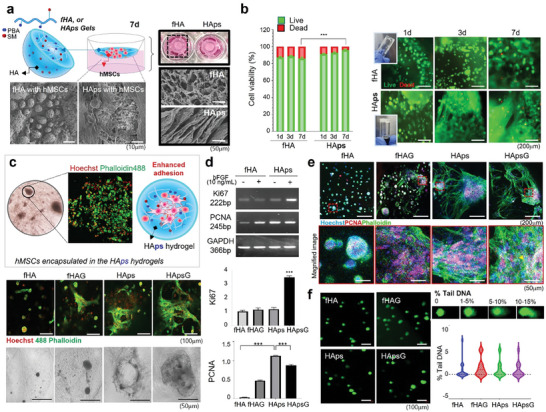
Evaluation of hMSC adhesion and proliferation in HAps hydrogel for 7 days. a) Scheme of the proliferation process of hMSCs encapsulated in HAps hydrogel in vitro. Photographic images of trans‐wells containing HAps hydrogels with hMSCs. SEM image of fHA and HAps with/without hMSCs. b) Viability of hMSCs cultured in hydrogels for 1, 3, and 7 d. The viability of hMSCs (%) from CCK‐8 assay and live/dead staining of hMSCs in fHA and HAps hydrogels. Scale bar = 100 µm. c) Phalloidin‐iFluor® 488 and Hoechst staining of hMSCs encapsulated in fHA and HAps hydrogels with and without bFGF after 7 days. Illustration of hMSC adhesion on HAps hydrogels and enhanced proliferation for tissue regeneration. fHAG and HApsG means fHA and HAps with growth factor, respectively. d) RT‐PCR images and quantitative RT‐PCR data from total RNA isolated from hMSCs encapsulated in the hydrogel showing the expression of PCNA and Ki‐67. e) Co‐localization of PCNA (green) and Hoechst (blue) of hMSCs grown in hydrogels after 7 d. PCNA shows proliferative ability and Phalloidin‐iFluor® 488 labels F‐actin structure. f) DNA ladder assay showing DNA fragmentation of hMSCs encapsulated in hydrogels. Comet assay was performed to measure the amount of unrepaired DNA damage in hydrogels. Tail moments (tail DNA % × length of tail) as quantified for each cell using Image J.

Subsequently, we cultured hMSCs within fHA and HAps hydrogels to observe their proliferation in these materials. When observing the behavior of hMSCs in the scaffold by scanning electron microscope (SEM) on day 7, cell adhesion and spreading were enhanced in HAps hydrogel compared to non‐crosslinked fHA polymer solution (**Figure** **3**a). In SEM images of fHA and HAps without cells, both had pores, but HAps pores showed more regular and elongated elliptical shapes with a uniform size (≈70–100 µm). Live/dead staining and CCK‐8 assays were performed to observe the proliferation of hMSCs in fHA and HAp in vitro. On days 1, 3, and 7, the cell viability of hMSCs in fHA was 87.34, 88.52, and 85.98%, respectively, and that in HAps was 91.15, 92.67, and 95.97%, respectively, according to the CCK‐8 assay (Figure 3b). In both the 3‐ and 7‐d groups, cell viability in HAps was higher compared to that in fHA. As shown in Figure 3c, this result is due to the encapsulated hMSCs within HAps effectively proliferating with enhanced adhesion and cell spreading, which was significantly observed in Alexa‐Fluor 488‐conjugated phalloidin F‐actin staining (Figure 3c). When analyzing the proliferation factors of hMSCs in 7‐d groups by RT‐PCR, the expression of PCNA, a proliferating cell nucleus antigen, and Ki‐67, a nuclear protein for cell proliferation, were significantly higher in HAps than in fHA (Figure 3d). PCNA immunostaining results (Figure 3e) also show that the expression of PCNA is remarkably increased in hMSCs encapsulated in HAps. These results were similarly observed in both HAps with and without basic fibroblast growth factor (bFGF). The fHA group indicated that the cells were in an environment in which cells could not spread, and some cells were lost. Comet assay was performed to evaluate DNA damage in hMSCs in fHA and HAps, and all groups showed negligible DNA damage (Figure 3f).

### 2.4 ECM Production and Mineralization from hMSCs in HAps Hydrogel After 14 Days

**Figure 4:**
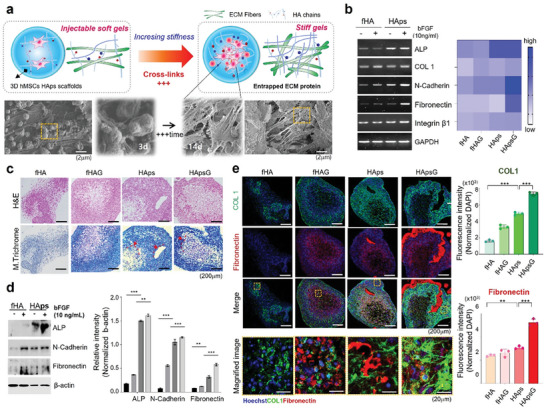
ECM production and mineralization from hMSCs in HAps hydrogel after 14 d. a) Illustration of stiffer hydrogel containing hMSCs and entrapping ECM proteins. SEM image of HAps with hMSCs over time. b) Expression of extracellular matrix‐related genes in hMSCs encapsulated in hydrogels by RT‐PCR and quantitative RT‐PCR. c) Hematoxylin & eosin (H&E) and MT staining images of hMSCs encapsulated in hydrogels. fHAG and HApsG means fHA and HAps with growth factor, respectively. d) Expression of ECM‐related protein in hMSCs encapsulated in hydrogels by western blotting. The protein expression of ALP, N‐cadherin, and fibronectin in HAps hydrogels was higher than in the fHA group. e) Immunofluorescence staining of Type 1 collagen (green) and fibronectin (red). The boxed region is an enlarged image of the hydrogels. The fHAG and HApsG hydrogels had been treated with bFGF. Quantitative analysis of type 1 collagen and fibronectin intensity. Nuclei were stained with Hoechst (blue). Scale bar, 100 µm.

As mentioned above, HAps hydrogel initially forms a soft and shear‐thinning hydrogel that can be injected by syringe, hardens gradually over time by self‐curing, and becomes stiff with a high storage modulus after 7 d. When we embedded hMSCs in HAps and grew them in 3D‐culture for 14 d, they spread and secreted ECM molecules. These molecules were entrapped in the stiffened hydrogel and interacted with the hMSCs. Then, the overall environment became similar to physiological tissue types (**Figure** **4**a). We performed RT‐PCR after 14 d of in vitro culture to investigate the ECM production of hMSCs adherent to HAps. When alkaline phosphatase (ALP), an enzyme involved in bone formation, is activated, the formation of new bone and mineralization processes occur constantly and repeatedly.^[^ ^22^ ^]^ The expression of these ALPs was higher in HAps than that in fHA (Figure 4b). In addition, in hMSCs in HAps, other factors containing COL1, a typical ECM marker protein, integrin in the cell membrane, fibronectin and N‐cadherin involved in the attachment of integrin and ECM, were highly expressed.^[^ ^23^ ^]^ We performed hematoxylin and eosin (H&E) staining for morphological analysis of hMSCs and Masson's trichrome (MT) staining to observe collagen and ECM in HAps (Figure 4c). Compared to the fHA group, it was remarkable that HAps and HAps‐G had a predominance of blue collagen and bone formation, and that there were many mature bones inside the bone. The quantitative increase in collagen and ECM was also evaluated by immunofluorescence staining and Western blot. When analyzing the expression of ALP, N‐cadherin, and fibronectin, the expression of ALP was observed more significantly in HAps groups, and N‐cadherin and fibronectin also highly increased in these groups. (Figure 4d). Immunostaining results showed a notable increase in COLI and fibronectin in the HAps group compared to the fHA group, and they further increased in the HAps‐G group (Figure 4e). These results show that ECM was effectively produced from hMSCs in HAps and that it deposited on the porous structure through smooth cell‐scaffold‐cell interaction. In addition, the increased stiffness of the hydrogel to which cells are attached is expected to affect the direction of stem cell differentiation, which is highly important in tissue regeneration.

### 2.5 Osteogenic Differentiation of hMSCs in HAps Hydrogel After 3 Weeks

**Figure 5:**
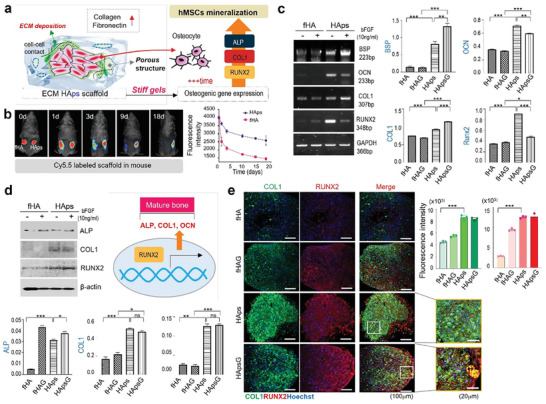
Osteogenic differentiation of hMSCs in HAps hydrogel after 3 weeks. a) Illustration of hMSCs mineralization by osteogenic gene expression. b) Degradation of HAps in mice observed by fluorescence image and quantitation. c) RT‐PCR analysis of mRNA levels of osteogenic related genes, BSP, OCN, Type 1 collagen, and RUNX2, and relative band intensity from RT‐PCR profiles. fHAG and HApsG means fHA and HAps with growth factor, respectively. d) Western blotting analysis of ALP, Type 1 collagen, and RUNX2 expression levels, and the band intensity quantified by normalized to respective β‐actin. e) Immunofluorescence staining of Type 1 collagen (green) and RUNX2 (red). The boxed region is an enlarged image of hydrogels. The fHAG and HApsG hydrogels had been treated with bFGF. Quantitative analysis of Type 1 collagen, and RUNX2 intensity.

Next, we evaluated how the increasing stiffness from the self‐curing process of HAps and ECM deposition affects osteogenesis by promoting hMSC proliferation and ALP activity, collagen synthesis, and bone mineralization within HAps after 4 weeks (**Figure** **5**a). When we subcutaneously injected Cy5.5‐fHA and Cy5.5‐HAps into mice and measured the resulting fluorescence intensity, more than half of Cy5.5 fluorescence remained in the stiff HAps hydrogel after 18 d in different with almost no fluorescence in fHA, showing its stability (Figure 5b). To observe the effect of HAps preventing the movement and loss of hMSCs into other site out of the target area, we subcutaneously injected hMSCs labelled with DiD fluorescence dye within or without HAps hydrogel (Figure S4, Supporting information). After 14 days, the significant differences in the fluorescence signal from hMSC was observed between the two groups showing the contribution of the hydrogel to the maintenance of hMSCs after injection. In vitro RT‐PCR was performed to analyze the degree of osteogenic differentiation of hMSCs seeded in HAps. In mRNA expression after 4 weeks, bone differentiation related markers, BSP, OCN, COL1, and RUNX2, were all significantly higher in HAps‐based groups (HAps, HApsG) compared to the fHA groups (fHA, fHAG).^[^ ^24^ ^]^ In HAps, OCN, a bone‐specific gene that promotes calcification, and RUNX2, an essential gene for bone formation, were expressed more than in HAps‐G (Figure 5c). Western blot analysis showed that COL1 and RUNX2 protein were highly expressed in HAps and HApsG, but there was no significant difference between the two groups (Figure 5d). In immunofluorescence staining, COL1 (green) and RUNX2 (red) were strongly detected in both HAps and HApsG, showing complete osteogenic differentiation at 4 weeks (Figure 5e). In particular, we observed the favorable changes in most factors in HAps were comparable to those in HApsG, so that we did not use bFGF in animal experiments. In addition, these positive effects in osteogenic differentiation were not observed in HAp without SM in histological analysis and immunofluorescence staining at 1‐, 2‐, and 4‐weeks showing the importance of the enhanced gelation by amine groups (Figure S5, Supporting information). Furthermore, in case of HAps samples, we observed intense staining with Alizarin Red S and von Kossa showing mineralization and calcium deposition in both in vitro 3D pellets and mouse tissues after subcutaneous injection (Figure S5,S6, Supporting information).

### 2.6 In vivo bone Regeneration of HAps Hydrogels Containing hMSCs in a Rat Femoral Defect Model

**Figure 6:**
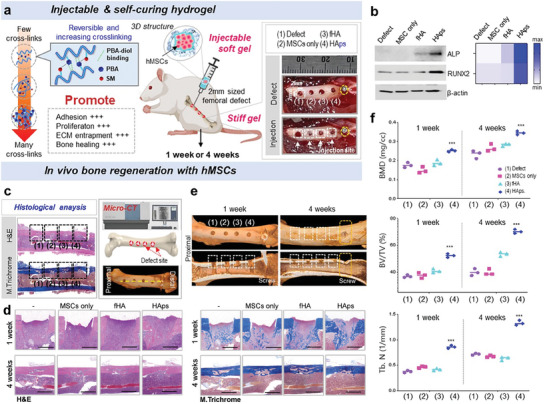
In vivo bone regeneration of HAps hydrogels containing hMSCs in a rat femoral defect model. a) Schematic diagram for HAps‐hydrogels containing hMSCs promoting in vivo bone regeneration. Photographic images of the transplantation of hMSCs in HAps hydrogel into the femoral defect of 8‐week‐old rats. b) Western blotting data after 1 week, showing the expression levels of bone differentiation markers, ALP and RUNX2. Quantitative protein levels of ALP, Type 1 collagen, and RUNX2 visualized by heatmap. c) Histological analysis of H&E and MT staining and micro‐CT image. d) Enlarged image of H&E and MT staining showing bone defect repair after 1 week and 4 weeks in defect model. e) Micro‐CT results of new bone formation in rat femur defect model after 1 week and 4 weeks. The treatment of 1) PBS; 2) hMSCs only; 3) hMSCs in fHA; 4) hMSCs in HAps. f) Quantitative results of micro‐CT evaluation 1 week, and 4 weeks after bone defect. The bone mineral density (BMD), the ratio of bone volume to total volume (BV/TV), and trabecular number (Tb.N) in rat femoral defect model.

The injectable hydrogel scaffolds are easy to administer into the body via syringe and are useful for regenerating tissues in the defect area with minimal damage. We expected that the shear‐thinning and self‐curing HAps would effectively promote tissue regeneration in the femoral defect model by enhancing stem cell adhesion, proliferation, and ECM entrapment (**Figure** **6**a). We made 2 × 2 × 2 mm defects at four locations on the femurs of 8‐week‐old rats. Subsequently, bone regeneration was monitored in four groups: defect only (control), hMSCs only, hMSCs in fHA, and hMSCs in HAps. Due to the shear‐thinning property of HAsp, there were no problems during its administration in vivo. In Western blotting data after 1 week, the expression levels of bone differentiation markers ALP and RUNX2 were significantly increased in HAps (Figure 6b). It showed that efficient adhesion and proliferation of hMSC in HAps resulted in effective bone regeneration. In particular, the expression level of ALP and RUNX2 was more than three times higher in HAps than in fHA, suggesting that the stiffening of HAps contributed to the promotion of osteogenic differentiation and mineral formation. Histological analysis and micro‐CT were also performed after 1 and 4 weeks to observe the regenerated bone (Figure 6c). H&E and MT staining data showed that HAps repaired the femoral defect by improving new bone formation at 1 week and promoted bone regeneration (Figure 6d). In the defect only and hMSCs only groups, no recovery was observed in the defect area. In HAps, the area of the femoral defect region was reduced, and the proliferation of osteocytes was observed in the femoral defect area (Figure 6e) and it was observed in both 2D and 3D CT images (Figure S7, Supporting information). Precise analysis of changes in BV/TV, BMD, and Tb.N was accomplished based on micro‐CT data (Figure 6f).^[^ ^25^ ^]^ The BMD after 1 week in the defect only, hMSCs only, hMSCs in fHA, and hMSCs in HAps were 0.163, 0.168, 0.175, and 0.257 g cm^−2^, respectively. The BMD in these groups after 4 weeks were 0.25, 0.259, 0.285, and 0.335 g cm^−2^, respectively, showing a significant elevation in only the HAps group. Similar to BMD, the BV/TV of HAps was 13 and 17% higher than those of fHA after 1 and 4 weeks, respectively. The Tb.N of HAps also showed a remarkable gap compared to all other groups. These results suggested that the injectable and self‐curing HAps significantly contributed to the overall bone recovery process using hMSCs by increasing bone density and influencing the mineralization process in the rat femoral defect model.

### 2.7 Discussion

A key feature in the crosslinking method of our HAps is the stabilization of the tetrahedral structure of the ionized PBA using SM. Ionized PBA is more reactive with diol compared to the free form of PBA. However, free form PBA is more stable at pH 7.4, so it is dominant under physiological conditions. Enhancement of ionized PBA using basic pH for stable gelation has been attempted in previous studies, but basic solutions are harmful to the human body. Lee et al. tried to change pH while maintaining the hydrogel properties, but it needs complicate process.^[^ ^26^ ^]^ Stabilization of ionized PBA using cationic charges was studied by Gao et al. at 2021.^[^ ^27^ ^]^ They showed that addition of cationic molecules like arginine or lysine could trigger gelation of PBA‐containing polymers that were in the sol state. With this consideration, in this study, we demonstrated that simple cationic modification of HA‐PBA enables gelation of the polymer solution. Cationic amine groups of SM with pKa values of 10.9, 8.4, and 9.9 can stabilize the anionic charge of ionized PBA and can increase its portion at neutral pH.^[^ ^28^ ^]^ Importantly, based on this mechanism, we developed a single‐component hydrogel of the HA‐PBA‐SM polymer solution without addition of other molecules. Using only one material for a hydrogel would be advantageous for simplification of the preparation process, quality control, and various tests essential for further clinical applications.

After administration of therapeutic stem cells into body, they spread throughout the body out of the disease area reducing the therapeutic efficacy. In addition, the survival rate is bound to drop as it must face a harsh internal environment. To overcome these limitations, in our study, stem cells were wrapped in hydrogel, which was initially in the form of a soft gel but quickly changed into a hard gel over time and used it as an injection material. This hydrogel is initially soft, so it can be injected into the defective area, and the stem cells wrapped in the gel actively migrate and proliferate within it. In addition, in these hydrogels, whose stiffness becomes stronger over time, stem cells are activated to differentiate into bone even without bioactive substances. Accordingly, this hydrogel maximizes the effect of tissue regeneration by creating an optimized in vivo microenvironment by regulating stem cell differentiation, cell migration, cell spreading, and adhesion.

We used HA as a backbone of our HAps hydrogel, but the original adhesion of HA to cells has been reported as poor. To overcome this challenge, researchers modified HA with biological ligands, like RGD peptide, for tissue engineering. In case of HAps, we observed vigorous adherence and spread of hMSCs onto the hydrogel without additional modification of biological molecules for adhesion. We expect that the cationic charges of SM would be beneficial for the adhesion of hMSCs as well as the stabilization of the ionized PBA and crosslinking. However, excessive cationic charges could destabilize the cell membrane and result in cytotoxicity, so that the number of cations must be carefully controlled.

ECM is a complex form of biological polymers containing various small molecules, and the interaction between cells and the surrounding ECM influences cell proliferation, differentiation, motility, and many phenotypes. In particular, it has been reported that physical parameters, such as the elastic modulus and pore size, of the ECM determine cell adhesion and morphology. In our experiments, we observed that hMSCs were stably attached and spread on HAps on the third day, and the adherent cells had interacted with HAps to form the ECM network on day 14. We expect that this progression is due to from multiple factors. First, we consider the high stiffness of HAsp after the self‐curing process and increased crosslinking. When tissue is regenerated by stem cells, it is necessary to adjust the scaffold as closely as possible to the biological microenvironment. Using a hydrogel that mimics a tissue‐like environment could effectively promote tissue regeneration.^[^ ^29^ ^]^ This phenomenon is particularly relevant considering that in this study, the applied tissue is bone. Second, hMSCs produce and secret molecules to form ECM over time, and the stable network of our HAps hydrogel could help entrap these molecules, preventing escape to other sites. In addition, HA, the basic material of the HAps hydrogel and ECM, is a glycosaminoglycan that does not induce an immune response.^[^ ^30^ ^]^ It is also useful for tissue engineering because it can interact with the cell surface and control cell proliferation and differentiation.^[^ ^31^ ^]^ Furthermore, as shown in Figure 5B, the HA backbone can degrade after a long time, so that newly regenerated ECM and tissue would ultimately fill in the area with no need to remove the HAsp hydrogel with additional treatment.

Recently, the application of various injectable hydrogels to bones as well as other musculoskeletal tissues like cartilages and discs showed their translational potential.^[^ ^32^ ^]^ These tissues are stiffer than other ones, so that the mechanical property of the hydrogel should be considered carefully. Therefore, as further studies, we are also planning to modulate the property of this hydrogel suitable for cartilage therapy. Considering clinical applications, the degradation rate of the hydrogel and the final products remained should be analyzed precisely to prevent long‐term toxicity in future. Practically, the limitation of this hydrogel comes from the self‐curing property. We observed that it occurred in aqueous condition right after the solubilization of the polymers, and the mechanical property of hydrogel changed over time. It was useful for bone therapy after injection in our study, but it could be a problem for the storage in liquid form. Preparing as a powder form lyophilized with cryo‐surfactants and enabling facile solubilization before using may solve this problem and would be useful for further application.

## 3 Conclusion

In summary, we developed an injectable, single‐component hydrogel with single‐component, HAps, and applied it as an hMSC scaffold for bone regeneration. The hydrogel was crosslinked by a reversible dynamic conjugation reaction between diol and ionized PBA, which was stabilized by amine groups. In particular, we demonstrated that simple cationic modification of PBA‐containing polymer enabled crosslinking and gelation at pH 7.4 without any additional materials. The chemical bonds are dynamic and reversible, providing the shear‐thinning property to the hydrogel for injection by syringe needles. Over time, self‐curing causes the hydrogel to become stiffer, which is another advantage of the HAps hydrogel. This hydrogel promotes proliferation and differentiation of stem cells in rigid tissue like bone. These properties were precisely evaluated in 3D culture in vitro, and all images and analysis of markers showed favorable results from hMSCs in HAps. Finally, the HAps‐hydrogel‐containing hMSCs were applied to a rat femoral defect model, and the results were analyzed by micro‐CT imaging and immunohistochemical staining. The overall results demonstrated the potential of HAps hydrogel as an hMSC scaffold for bone regeneration, and we expect that its useful properties could also be leveraged in broad applications in stem cell therapy and tissue engineering.

## 4 Experimental Section

### Materials

Hyaluronic acid sodium salt (HA) (1 MDa) was purchased from Lifecore (Chaska, MN, USA). The 4‐(4,6‐Dimethoxy1,3,5‐triazin‐2‐yl)−4‐methylmorpholinium chloride (DMTMM), spermidine (SM), (3‐Aminomethylphenyl) boronic acid (PBA), β‐glycerophosphate and dimethyl sulfoxide (DMSO) were purchased from Sigma–Aldrich (St. Louis, MO, USA). Human mesenchymal stem cells (hMSCs) were purchased from Lonza (Basel, Switzerland). DMEM high glucose media and Dulbecco's phosphate‐buffered saline (DPBS) were purchased from Biowest (Riverside, MO, USA). Antibodies and Hoechst were purchased from Invitrogen (Waltham, MA, USA). Sulfo‐cy5.5‐amine was purchased from Lumiprobe (Cockeysville, MD, USA). Basic fibroblast growth factor (bFGF) proteins were purchased from R&D Systems (Minneapolis, MN, USA). Alexa‐Fluor 488‐conjugated phalloidin was purchased from Thermo Fisher Scientific (Waltham, MA, USA). About abbreviations, the unsynthesized HA hydrogel was named free HA (fHA), the HA hydrogel conjugated with PBA was named HAp hydrogel, and the HA hydrogel conjugated with PBA and SM was named HAps. In addition, the samples with growth factors were named fHAG and HApsG, respectively.

### Synthesis of the Hyaluronic Acid Modified with Phenylboronic Acid and Spermidine (HAps)

HA modified with PBA (HAp) was prepared via amide coupling reaction. In brief, 40 mg of HA and DMTMM (100 mg, 0.42 mmol) were dissolved in 5 mL of PBS buffer and stirred for 10 min. Then, PBA (20 mg, 0.11 mmol) was added to the solution, and the pH was adjusted to approximately 7.0 with 1 m NaOH. After incubation for 72 h at room temperature, the solution was dialyzed against DI water with a molecular weight cut‐off of 6000–8000 Da for 24 h. Subsequently, the reaction product was freeze‐dried, and a white sponge‐like product was obtained. The reaction product was assayed by the alizarin reaction.

For HAps, 20 mg of HA‐mPBA and DMTMM (30 mg, 0.13 mmol) were dissolved in 5 mL of DI water for 30 min. Then, spermidine (15 mg, 0.11 mmol) was added to the mixture and stirred for 24 h and dialyzed against deionized (DI) water for 24 h. HAps was finally obtained after lyophilization.

For the degradation study in vivo, HAps was labelled with Cy5.5. In brief, HA (40 mg, 0.1055 mmol), DMTMM (30 mg, 0.11 mmol) and sulfo‐cy5.5‐amine (0.07 mg, 0.09 µmol) were dissolved in a DI water. After 24 h, this solution was dialyzed against DI water for 24 h, and cy5.5‐labeled HA was obtained after lyophilization.

### Rheology Test of HAps

Rheology was analyzed using a DHR10 rheometer (TA Instruments, USA). A 20‐mm parallel plate was used to perform rotation and oscillation tests. The hydrogel was placed on the bottom plate and analyzed in oscillation and rotation modes. In oscillation mode, the storage modulus was analyzed with increasing frequency. This mode was also used to measure tan(delta), phase angle as a function of oscillation strain, change in complex modulus as a function of oscillation, and stress relaxation as a function of time. The reversible transition of the gel was measured using a 1–200% strain change in the same mode. The rotation mode was used to measure the viscosity of hydrogel.

### Cell Culture and In Vitro Assays

hMSCs (Lonza, Walkersville, USA) isolated from the bone marrow is used. They were cultured in DMEM high glucose supplemented with 10% FBS and 1% antibiotic‐antimycotic. The culture medium was replaced every 3 d, and all experiments were performed with cells at passages 7. The stabilized cells were harvested with trypsin‐EDTA and centrifuged at 1300 rpm for 2 min before use. After 3D culture of hMSCs in hydrogel on trans‐well plates for a predetermined period, live/dead staining and CCK‐8 and Comet assays were performed according to the manufacturer's protocols. The fluorescence images were obtained using a LSM800 w/Airyscan and Axioimager M1 (Carl Zeiss, Oberkochen, Germany).

### 3D Culture of hMSCs in HAps Hydrogels

hMSCs (Lonza, Walkersville, USA) at passage 7 were used in this study. Briefly, hMSCs were resuspended and then loaded into the insert chamber of the Transwell system at a density of 3 × 10^6^ cells/HAps hydrogels (100 µL). Afterwards, Live/dead staining and CCK‐8 were performed according to the manufacturer's protocols. Also, to detect DNA damage, 2 × 10^5^ hMSCs were collected from the hMSC‐encapsulated HAps, and aliquots were mixed with 50 µL of low‐melting point agarose at 37 °C and spread on slides to solidify. A total of 30 comets randomly captured on each slide were examined at 400× magnification using a LSM800 confocal microscope (Carl Zeiss, Oberkochen, Germany). To quantify DNA damage, tail intensity and tail moment were recorded and then statistically analyzed by *t*‐test.

### In Vivo Experiments

The animal study was approved by the Institutional Animal Care and Use Committee (IACUC) of The Catholic University of Korea (Approval No. 2022‐0343‐03 for mice and 2022‐0199‐03 for rats). For the in vivo degradation study, cy5.5‐lablled fHA and HAps were subcutaneously injected into the back of CD1 mice after shaving, and the resulting fluorescence intensity was observed for 30 d using Amersham ImageQuant 800. For modeling a femoral defect, 8‐week‐old SD rats were purchased from Orient Bio (Seongnam, Korea). Holes with dimensions of 2 × 2 × 2 mm were drilled in the femur while rats were anesthetized with 2% isoflurane, and hMSCs in hydrogel were injected into the hole site.

### Micro‐CT Imaging

At 1 and 4 weeks after surgery, rats were sacrificed, and the femur bone was harvested and fixed with 4% paraformaldehyde. Subsequently, Micro‐CT (SKYSCAN1176, BRUKER, Massachusetts, USA) was used to analyze the degree of bone regeneration. Bone volume (BV), bone mineral density (BMD), and trabecular number (Tb.N) were analyzed with the analysis software of Micro‐CT device.

### Histology Analysis

Paraffin blocks were prepared for histological analysis. Hematoxylin and eosin (H&E) and Masson's trichrome (MT) staining were performed following deparaffinization and rehydration. Samples were observed using a Nikon ECLIPSE Ti2 (Nikon, Tokyo, Japan).

### Statistical Analysis

Statistical analysis was performed using the Student's *t*‐test with SigmaPlot software. *P < 0.05, **P < 0.01, and ***P < 0.001 were considered as statistically significant.

## Conflict of Interest

The authors declare no conflict of interest.

## Supporting information

Supporting Information

## Data Availability

The data that support the findings of this study are available from the corresponding author upon reasonable request.
