# Supplementary material for: Injectable and Self‐Curing Single‐Component Hydrogel for Stem Cell Encapsulation and In Vivo Bone Regeneration
Source: Adv Sci (Weinh). 2024 Feb 14;11(16):2304861. doi: 10.1002/advs.202304861 (PMC11040337; doi:10.1002/advs.202304861)
Supplement: Supplementary file 1 — Supporting Information [file ADVS-11-2304861-s001.pdf]

ADVANCED SCIENCE
Open Access

Supporting Information

for *Adv. Sci.*, DOI 10.1002/advs.202304861

Injectable and Self-Curing Single-Component Hydrogel for Stem Cell Encapsulation and In Vivo Bone Regeneration

*Seo Young Cheon, Ji Sun Park, Yeeun Lee, Chaehyun Lee, Hayoung Jeon, Donghyun Lee, Se Hee Kim, Seong Gi Lim and Heebeom Koo**

Supporting Information

Injectable and self-curing single-component hydrogel for stem cell encapsulation and *in vivo* bone regeneration

Seo Young Cheon[1], Ji Sun Park[1], Yeeun Lee, Chaehyun Lee, Hayoung Jeon, Donghyun Lee, Se Hee Kim, Seong Gi Lim, Heebeom Koo*

Department of Medical Life Sciences, Department of Biomedicine & Health Sciences, and Catholic Photomedicine Research Institute, College of Medicine, The Catholic University of Korea, 222 Banpo-daero, Seocho-gu, Seoul, 06591, Republic of Korea

*Corresponding author: Department of Medical Life Sciences, College of Medicine, The Catholic University of Korea. 222 Banpo-daero, Seocho-gu, Seoul, 06591, Republic of Korea

E-mail addresses: hbkoo@catholic.ac.kr (H. Koo).

[1]These authors contributed equally to this work.

Table S1. The degree of substitution of PBA in hyaluronic acid evaluated by alizarin red S assay

sample No.	Amide coupling reaction condition		DS (%)
	Reaction time (h)	HA:PBA:DMTMM (mmol:mmol:mmol)	
1	48	1:1:4	31
2	72	1:1:4	35
3	48	1:2:4	33
4	72	1:2:4	35
5	48	1:3:4	33
6	72	1:3:4	34

Table S2. Photo-images of HAps hydrogel from various reaction condition between HA-PBA and spermidine

Sample No.	1	2	3
HA-PBA:spermidine (molar ratio)	1:2	1:2	1:3
Reaction time (h)	24	36	24
Gelation image	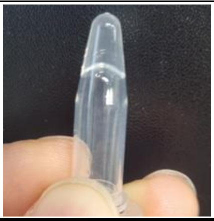	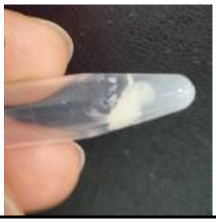	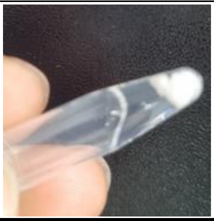

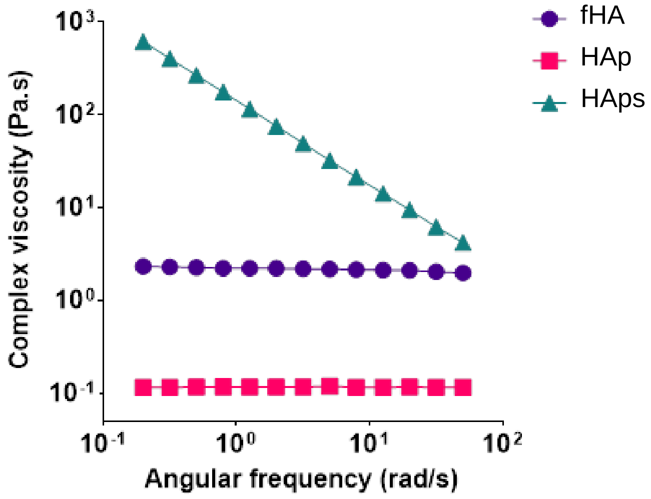

Figure S1. Rheology result of complex viscosity- angular frequency

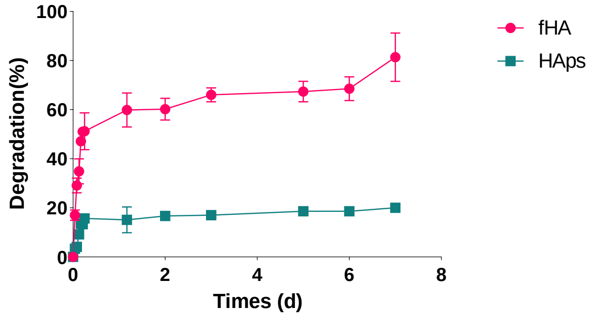

Figure S2. *In vitro* degradation of free HA hydrogel (fHA) and HA-PBA-SM hydrogels (HAps) by hyaluronidase.

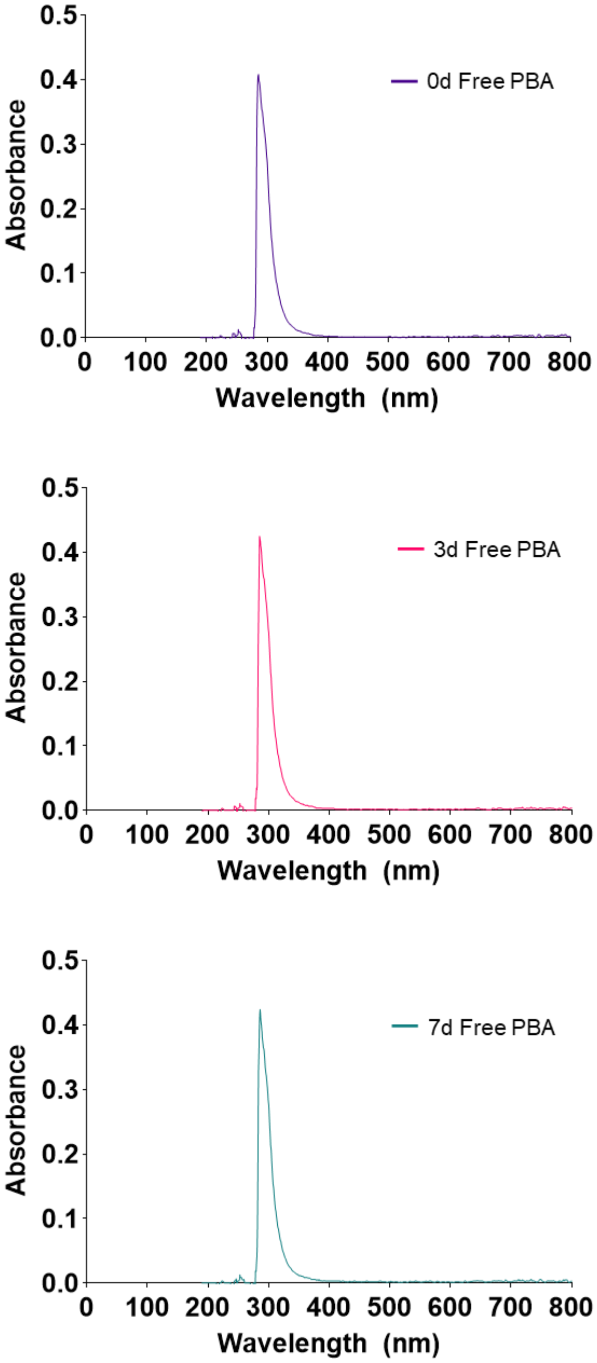

Figure S3. UV-vis spectra of free PBA over time.

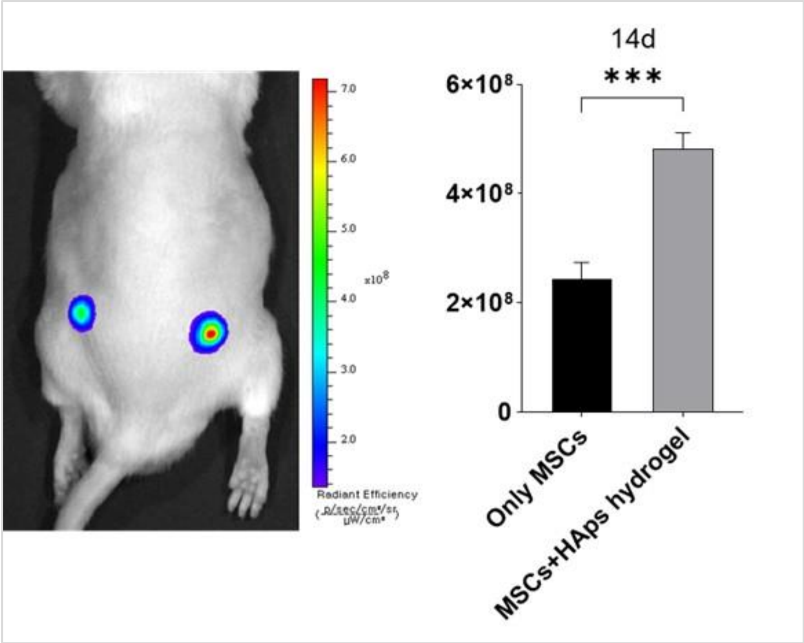

Figure S4. Evaluation of hMSCs maintenance according to the presence or absence of hydrogel *in vivo.*

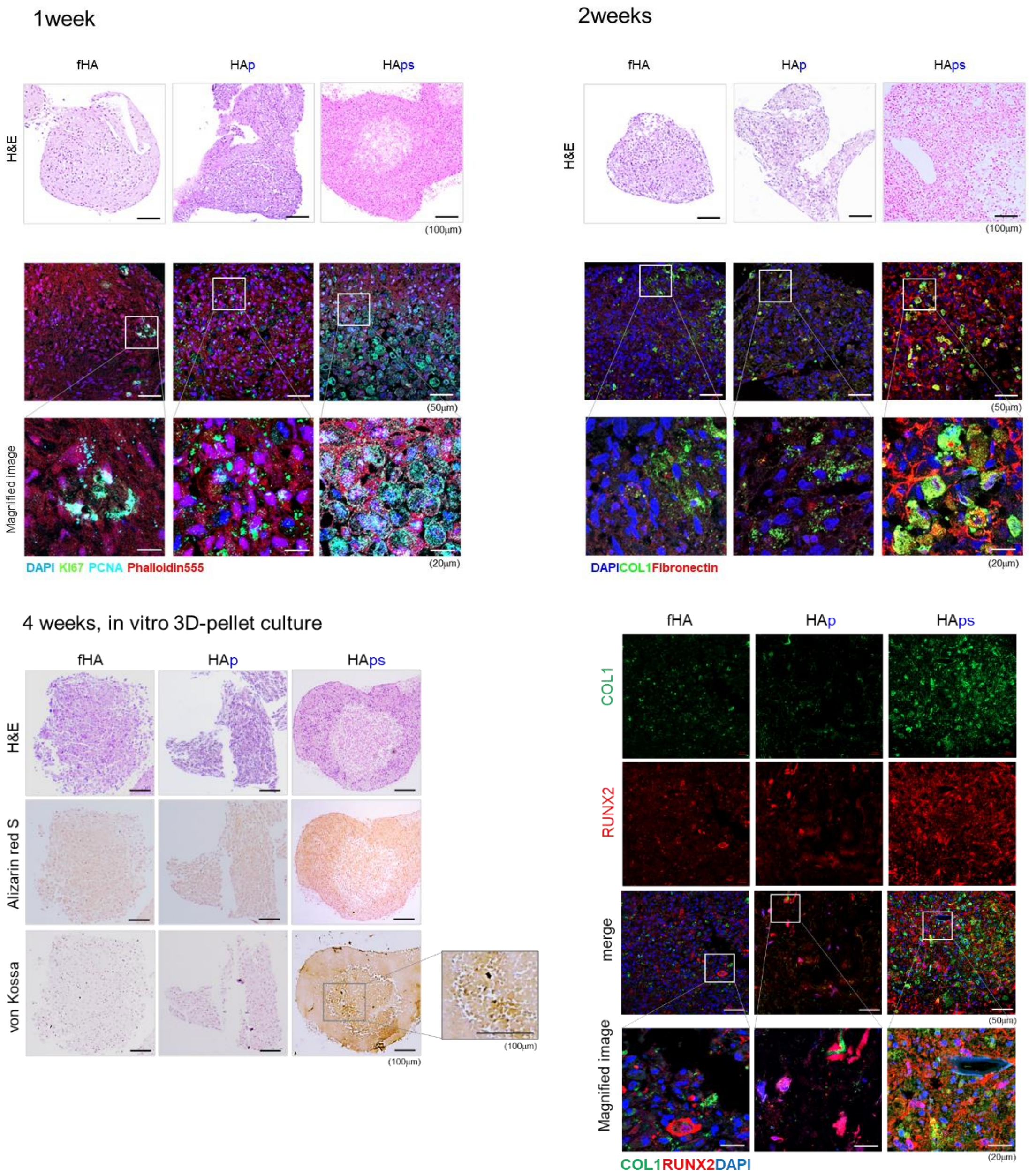

Figure S5. Histological analysis and immunofluorescence staining of hMSCs embedded fHA, HAp, and HAps *in vitro* at 1-, 2-, and 4-weeks.

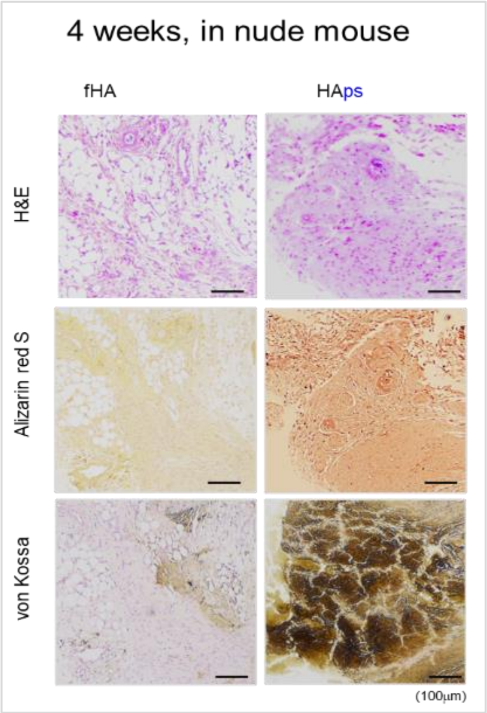

Figure S6. Alizarin Red S, and von Kossa staining of osteogenic differentiation of hMSCs embedded fHA, HAps *in vivo* for 4 weeks.

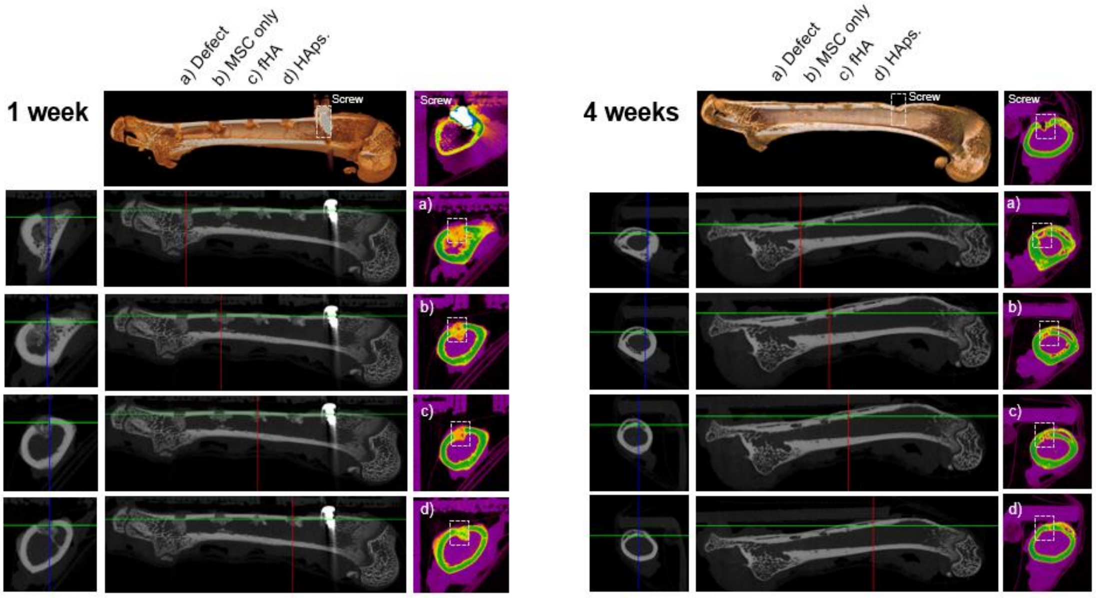

Figure S7. Micro-CT analysis of femoral defects after a 1-, 4-week treatment with hydrogels. Axial, lateral, two-dimensional (2D) and 3D reconstructed micro-CT images of bone at the femoral.
